# Stress and stressors of medical student near-peer tutors during courses: a psychophysiological mixed methods study

**DOI:** 10.1186/s12909-019-1521-2

**Published:** 2019-04-02

**Authors:** Jan Hundertmark, Simone Alvarez, Svetla Loukanova, Jobst-Hendrik Schultz

**Affiliations:** 1 Clinic for General Internal Medicine and Psychosomatics, University Hospital Heidelberg, Im Neuenheimer Feld 410, 69120 Heidelberg, Germany; 2 Department of General Practice and Health Services Research, University Hospital Heidelberg, Im Neuenheimer Feld 130.3, 69120 Heidelberg, Germany

**Keywords:** Peer assisted learning, Peer teaching, Tutors, Stress, Medical students, Cortisol, Heart rate variability, Content analysis, Mixed methods

## Abstract

**Background:**

Structured peer-led tutorial courses are widespread and indispensable teaching methods that relieve teaching staff and contribute to the development of students’ competencies. Nevertheless, despite high general stress levels in medical students and associated increases in psychopathology, specific knowledge of peer tutors’ additional burdens is very limited.

**Methods:**

Sixty student near-peer tutors from two structured peer-teaching programmes volunteered to participate. On multiple occasions in three different course sessions, we assessed tutors’ subjective stress, affective state, heart rate variability, and salivary cortisol. Additionally, tutors named everyday and course-specific stressors, which were evaluated by means of content analyses.

**Results:**

The study participation rate was high (63% of all active tutors). The participating tutors are socially well adapted and resilient individuals. They report a variety of stressors such as time pressure, participant characteristics, teacher role demands, and study requirements, but nevertheless display only moderate psychological and physiological stress that decreases over sessions. Tutors’ negative affect in sessions is low; their positive affect is consistently high for senior as well as novice tutors. Tutors rate their courses’ quality as high and quickly recover after sessions.

**Conclusions:**

Tutors successfully cope with teaching-associated and everyday life demands. The results corroborate the viability and success of current peer-teaching programmes from the tutors’ perspective. This study is the first to comprehensively quantify tutors’ stress and describe frequent stressors, thus contributing to the development of better peer teaching programmes and tutor qualification training.

**Electronic supplementary material:**

The online version of this article (10.1186/s12909-019-1521-2) contains supplementary material, which is available to authorized users.

## Background

Peer teaching, commonly understood as educational arrangements in which a student teaches one or more fellow students [1], has a long history in both medical and academic education in general [2, 3]. It is generally perceived as an effective, feasible, and cost-efficient learning method that alleviates demands on clinical instructors and improves overall clinical experiences for students [4–7].

One important and prevalent near-peer teaching setting is structured peer-led tutorial courses, in which experienced and trained students (student peer or near-peer tutors, hereinafter referred to as tutors) instruct small groups in subjects such as communication and anatomy or in clinical skills [1, 8, 9]. These tutorial courses have, if properly implemented, the potential to be as effective as conventional staff-led classes [6, 10–13]. Tutors are usually highly motivated and engaged [14, 15] and possess high cognitive and social congruence [16]: Their awareness and familiarity with their tutees’ current level of knowledge and study situation allows them to adapt their use of didactics, language, and exercise difficulty; similarities in age, life situation, and qualification result in a more personal and amicable learning atmosphere with less fear or shame of asking questions or making mistakes. Tutors themselves also benefit from their work, apart from mere financial compensation. They usually undergo some form of selection or training process [17, 18] and gain communication, teaching, and clinical skills that can lead to improved grades, patient interaction, learning behaviour, self-reflection, and even personal resilience [19–24].

Notwithstanding these apparent benefits, tutorship is time-consuming and places additional demands and responsibilities on tutors, including leadership, prioritisation, and identifying and coping with own mistakes or weaknesses [24]. Despite the growing prevalence of structured peer-led tutorial courses, researchers have paid little attention to these additional challenges. This is especially surprising against the background of medical students’ high stress levels [25–27].

Stress can be defined as an organism’s response to certain triggering factors that result from interaction with the environment [28]. It aims at enabling individuals to perceive and cope with threats and challenges [29]. Stress encompasses non-specific sympathetic activation, leading to increased heart rate and blood pressure, the release of catecholamines and corticosteroids, along with psychological changes like heightened alertness or increased attention [30], as well as other fine-tuned, individualised response patterns [31]. In moderate levels, stress can be a source of motivation and enhance learning [32]; therefore, it can be beneficial for medical training [33, 34]. However, excessive stress can lead to physical, emotional, and mental health problems [35] and is associated with an increased prevalence of psychopathology in medical students, such as anxiety, burnout, depression, and substance abuse [26, 36–40]. For instance, 30 to 40% of medical students experience relevant psychopathological symptoms [41]. Similarly, Koehl-Hackert et al. [42] found a burnout prevalence of 20% in German final year medical students.

Stress stems from environmental events, commonly referred to as stressors, that are appraised by a person as taxing or exceeding his or her resources [43, 44]. Typical stressors reported by medical students are information overload, financial debt, lack of leisure time, curriculum and course organisation, work-related pressures including work relationships and career choices [45]. The diverse requirements of tutoring activities, such as preparing teaching materials, giving presentations, leading a group, resolving in-group conflicts, and completing compulsory training, potentially add further burdens to this array; however, whether these requirements are experienced as stimulating or harmful strongly depends on tutors’ individual resources and coping capacities [28, 44].

This study focusses on two structured near-peer-teaching programmes at Heidelberg Medical Faculty: “Anatomie am Lebenden plus” [“living anatomy plus”] and abdominal ultrasonography. Anatomie am Lebenden plus (AaL^*plus*^) consists of sequential two-week practical seminars which are mandatory in all four preclinical semesters [46]. Over five sessions, students acquire basic physical examination techniques, take medical histories of standardised simulation patients [47], and approach and solve medical cases using problem-based learning methods [48]. Aal^*plus*^ tutorial courses have ten to twelve participants and are taught by a team of two tutors, typically a novice and a senior tutor. Abdominal ultrasonography is a highly popular one-week elective in which preclinical students learn to handle ultrasonic devices, locate standard scan planes, perform basic clinical ultrasound tests, and identify pathologies or abnormalities. Tutors teach groups of five participants for two hours each day. Senior tutors work alone, whereas novice tutors are obliged to observe a senior tutor’s course session before teaching the same unit himself under that senior tutor’s supervision. In both AaL^*plus*^ and sonography, tutors follow a set thematic structure; however, they are required to prepare study material, revise and present course content, answer participants’ questions, give feedback, moderate practice phases, and handle group dynamics. Teaching an AaL^*plus*^ course takes a tutor about 8 h plus about 9 h of preparation in total; sonography courses require about 15 h plus about 10 h of preparation. Tutors typically take on two to four (AaL^*plus*^) or one or two (sonography) courses per semester. Moreover, the compulsory preceding qualification places additional burdens on tutors’ time: They need to complete 40 to 50 h of unpaid didactical, communicational, and technical training before being allowed to teach. All AaL^*plus*^ and sonography courses take place in the late afternoons and evening.

Taken together, tutor programmes offer a multitude of potential teaching benefits, but may place considerable additional demands on tutors selected from the already burdened population of medical students; however, the extent or nature of tutors’ specific stress has not yet been systematically investigated. This study aims to fill this research gap by comprehensively assessing tutors’ stressors and psychophysiological stress responses in a naturalistic setting. In addition to this descriptive assessment, we hypothesised that unexperienced (novice) tutors experience more stress than experienced (senior) tutors, that stress levels drop over course days, and that certain personality traits (extraversion, low neuroticism, resilience, low perfectionism, and secure attachment) are negatively correlated with tutor stress.

## Methods

### Participants

We invited all active tutors from the AaL^*plus*^ and sonography programmes to participate. 36 AaL^*plus*^ tutors (17 novice, 19 senior tutors, 17 female, 19 male; out of 53 AaL^*plus*^ active tutors in total, participation rate 68%) and 24 sonography tutors (11 novice, 13 senior tutors, 15 female, 9 male; out of 42 active sonography tutors in total, participation rate 57%) volunteered. Mean age was 22.8 years (*SD* = 2.6); senior tutors were on average 1.4 years (*SD* = 2.5) older and had already taught 7.0 (*SD* = 6.0) courses. In addition to the given time requirements for teaching, study participation took tutors an extra three hours, for which they received financial compensation.

### Procedure

**Fig. 1:**
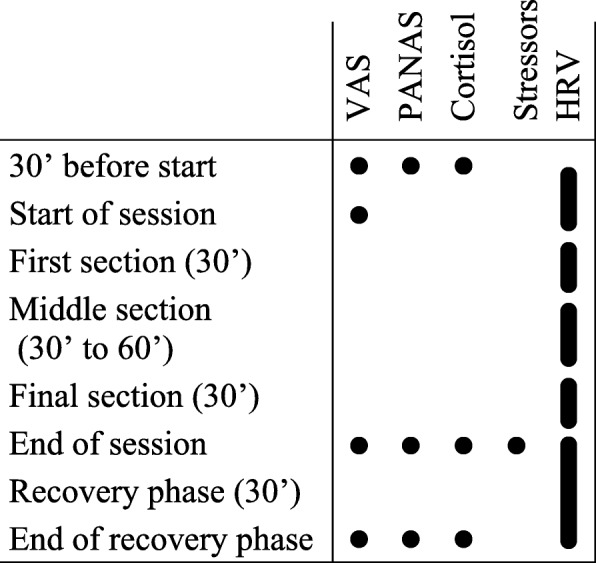
Overview on measurement points/periods on all three survey days. Abbreviations: see Instruments section

Recruitment and data collection took place in winter semester 2016/2017. At an initial session, participants were informed about the study procedure, gave their written consent, completed a first set of personality questionnaires, and assigned one of their tutorial courses for data collection. The actual stress assessment took place on days 1, 2, and 4 of the respective course. For AaL^*plus*^, this included sessions on history taking, physical examination (neurological or locomotor system), and problem-based learning. For sonography, the respective session topics were aorta and vena cava, liver and gall bladder, and kidney and urinary bladder. On each survey day, data collection followed the same routine, as depicted in Fig. 1. An investigator was present at all times to assist tutors with study procedures, saliva samples, and the equipment of the heart rate monitors, which had to be worn during a course session’s whole measurement period. Participants arrived 30 min before the beginning of their respective session, started heart rate recording, completed the first set of measures, and continued session preparation as usual. An extensive assessment followed directly after teaching, including written reports of stressors experienced in the respective session. Subsequently, participants entered a 30-min recovery phase. We instructed them to remain in silence and relax in a sitting position. They were free to think about anything and let their minds wander, but should not engage in mental work like planning their day or revising any study matter. Afterwards, participants completed a final assessment and stopped heart rate recording. Following the last survey day, participants additionally reported every-day stressors and were conclusively debriefed.

### Design

This study followed a mixed-methods design, combining quantitative and qualitative research methods and both psychological and physiological data. We assessed the extent and time profiles of tutors’ stress using repeated quantitative measures before, during, and after tutorial sessions; moreover, the subsequent half-hour rest period served to assess the extent and speed of psychophysiological recovery. The employed psychological measures are subjective stress (VAS-Stress; for abbreviations: see Instruments section), positive and negative affect (PANAS), and written reports of relevant stressors during and outside of courses; the physiological measures are salivary cortisol and heart rate variability (HRV). To investigate potential protective or risk factors, we assessed selected aspects of participants’ personality using validated questionnaires.

### Instruments

#### VAS-stress

Visual analogue scales (VAS) are continuous, easy-to-use one-item interval scales that allow self-assessments of internal feelings, perceptions, or sensations that are difficult to phrase or measure on scales with predetermined intervals. VAS are typically composed of a 100 mm horizontal line on which subjects can rate their current mental state, with end anchors such as *not at all stressed* and *extremely stressed* in this study [49, 50]. Its validity, reliability, and sensitivity to change have been shown for various scopes of application, including subjective stress [51, 52]. The VAS-Stress’s theoretical scale range is 0 to 10. In this study, participants further used a similar scale to rate the subjective quality of their respective courses.

#### PANAS

Positive and negative affect are the two dominant dimensions of emotional experience [53, 54]. In this study, we used the established Positive And Negative Affect Schedule’s (PANAS) short version. The PANAS measures a person’s current affective state using 5-level Likert scales on two dimensions consisting of five items each [55, 56]: active, determined, attentive, inspired, and alert for Positive Affect (PA) and afraid, nervous, upset, hostile and ashamed for Negative Affect (NA). The PANAS’s theoretical scale range is 1 to 5.

#### Salivary cortisol

Due to its sensitivity to physical or psychological stress as well as its easy traceability in saliva, cortisol has been the most widely used biomarker in stress studies for decades [57–59]. As effector hormone of the hypothalamic-pituitary-adrenocortical (HPA) axis, which mediates slower, but more enduring aspects of the stress response, it has wide-ranging effects on the whole body [60–63]. Cortisol level increases become peripherally detectable only five minutes after the stressor and reach their maximum ten to 30 min after stressor cessation [64]. Salivary cortisol is highly correlated with blood cortisol levels [65–67], especially with the free cortisol fraction that is able to cross the blood-brain barrier and bind to receptors at structures responsible for high-level cognitive functions such as learning, memory, and emotion processing [64, 68, 69].

In this study, we used synthetic fibre swabs (Salivetten® by Sarstedt) as the most convenient, economic, and valid method of saliva collection [67, 70]. Since cortisol levels react to physical activity as well as glucose and drug intake [71–77], we instructed participants to strictly refrain from workout, the consumption of alcohol, caffeine, and nicotine on survey days, as well as eating 30 min and drinking five minutes before taking samples [78]. All saliva samples were collected after sessions, deep-frozen, and analysed in the Heidelberg University Hospital’s Steroid Lab.

Cortisol levels follow a pronounced circadian rhythm, with a sharp rise after awakening and a linear decline from afternoon to evening [64, 79], which needs to be taken into account when using cortisol as a stress marker. In this study, participants autonomously took three (AaL^*plus*^) or four (sonography) saliva samples at specified times on the late afternoon and evening of a rest day, usually Sunday, following the same instructions for sample taking as on other survey days. The resulting sample values allowed us to model linear time profiles of participants’ normal cortisol levels, which then served as a baseline against which we compared participants’ cortisol levels before and after courses.

#### Heart rate variability

Heart rate variability (HRV) is the physiological phenomenon of varying time intervals between heartbeats even at constant output or resting conditions. HRV is a non-invasive, reliable, and valid index of cardiac vagal tone and hence parasympathetic activity [80]; it reflects the heart’s capacity to adapt to changing circumstances and unforeseen stimuli [81]. Independent associations of vagally mediated HRV with general mortality and morbidity as well as work environment stressors have been consistently shown [82–86]. Short term HRV analyses, as used in this study, reflect the autonomic balance shift during acute mental stress, comprising increased sympathetic arousal and parasympathetic withdrawal.

Modern heart rate monitors usually consist of wireless chest strap electrodes and additional equipment, e.g. a watch, for data storage. They provide researchers with an affordable, robust, reliable, and highly valid means to record HRV data [87, 88]. HRV analysis software is able to determine common time domain and frequency domain measures [86, 89]. In this study, we used Polar V800® sports watches, a Microsoft Excel® macro [90] for data transformation, and the Kubios HRV 2.2® analysis software [91] with medium-level artefact correction settings and the smoothing-priors de-trending method [92]. The root mean square of successive differences in interbeat intervals (RMSSD) [86, 89] served as the primary HRV measure. The RMSSD is largely independent from the measurement period’s duration [93]; however, we chose a standardised length of 30 min for analysis wherever possible (see Fig. 1).

#### Participant characteristics

For further correlational analyses, we assessed participants’ personality, perfectionism, resilience, and attachment style before the start of the study. We employed the German 21-item short version of the Big Five Inventory (BFI-K) for assessing personality [94–96]; for perfectionism, the German 8-item short version of the Revised Almost Perfect Scale (SAPS-R, subscales *standards*, i.e. high performance expectations, and *discrepancy*, i.e. self-critical performance evaluations) [97, 98]; for resilience, the German 13-item short version of the Resilience Scale (RS 13, subscales *personal competence* and *acceptance of self and life*) [99, 100]; and for attachment style, the 16-item German version of the Adult Attachment Scale (AAS, subscales *Depend*, indicating ability to trust in others and depend on them to be available when needed, *Anxiety* in relationships, and *Close*, indicating ability to be comfortable with closeness and intimacy) [101, 102]. Additionally, we measured chronic stress with the German version of the Trier Inventory for the Assessment of Chronic Stress (TICS), a 57-item 10-scales questionnaire including one screening scale [103]. All instruments possess satisfactory to excellent psychometric criteria and have been validated in numerous studies (for further references see [94–103]).

### Data analysis

Prior to data analysis, we performed a thorough data screening, including checks for normality, linearity, floor effects, and outliers, as well as independence and homogeneity between measures. We detected no significant outliers, limitations, or violations of the general linear model assumptions, except for slightly skewed cortisol level distributions; however, the analysis methods used in this study are generally seen as sufficiently robust [104, 105].

To adjust cortisol values for measurement time and participants’ individual circadian rhythms, we first determined baseline cortisol levels from participants’ rest day cortisol samples by means of hierarchical linear modelling (HLM) [106]. HLM is an extension of linear regression modelling for hierarchical data structures, such as observations nested in participants, that should be employed if the dataset’s intraclass correlation (ICC) is above .05. We first computed a restricted maximum likelihood estimation model with measurement *time* as main predictor and compared it to a null model without predictors. With the resulting model, we then calculated individual time-dependent cortisol reference values, against which we next compared the respective participants’ cortisol levels at the different measurement points on survey days. The final net cortisol values represent the teaching-specific HPA axis activation.

We analysed participants’ quantitative stress profiles (VAS-Stress, PA, NA, net cortisol, and HRV) with repeated-measures analyses of variances (RM-ANOVAs), which is used to assess several or repeated measurements of the same participants under different conditions. Given this method’s sensitivity to missing values, we imputed lost data (2.4% of all data, no indications of non-random missing patterns) using the expectation maximisation procedure [107] before running the RM-ANOVAs. Afterwards, we also tested for violations of the sphericity of repeated measures assumption using Mauchly’s test and, where required, corrected the analyses’ degrees of freedom using the Huynh-Feldt procedure. Finally, we investigated the potential influence of tutorship experience as well as protective personality factors by means of covariate analyses.

We used the statistic package R 3.3.2 with the lme4 library for HLM and SPSS 25.0 for all other purposes, with a general significance level of α = .05. As to qualitative data, we performed three content analyses with tutors’ written reports on their course-specific (AaL^*plus*^ and sonography) and everday life stressors. In these analyses, one researcher (JH) clustered participants’ statements into theme groups to create inductive category systems [108].

## Results

### Sample characteristics

As multiple *t*-tests revealed that participant characteristics did not differ depending on course type (*AaL*^plus^ and *sonography*), tutor experience (*senior* and *novice* tutors), age, or gender, we aggregated descriptive data over these factors. Comparisons of participant characteristics with respective norm values indicate that tutors are mostly outgoing, secure, competent and conscientious individuals with a balanced lifestyle. Their reports on chronic stress do not differ significantly from general population values, except for slightly higher pressure to perform and lower social overload. Additional file 1: Table S1 within the online supplementary material gives a comprehensive overview on participants’ descriptive data as well as norm sample values and comparisons.

### Psychological stress

**Fig. 2:**
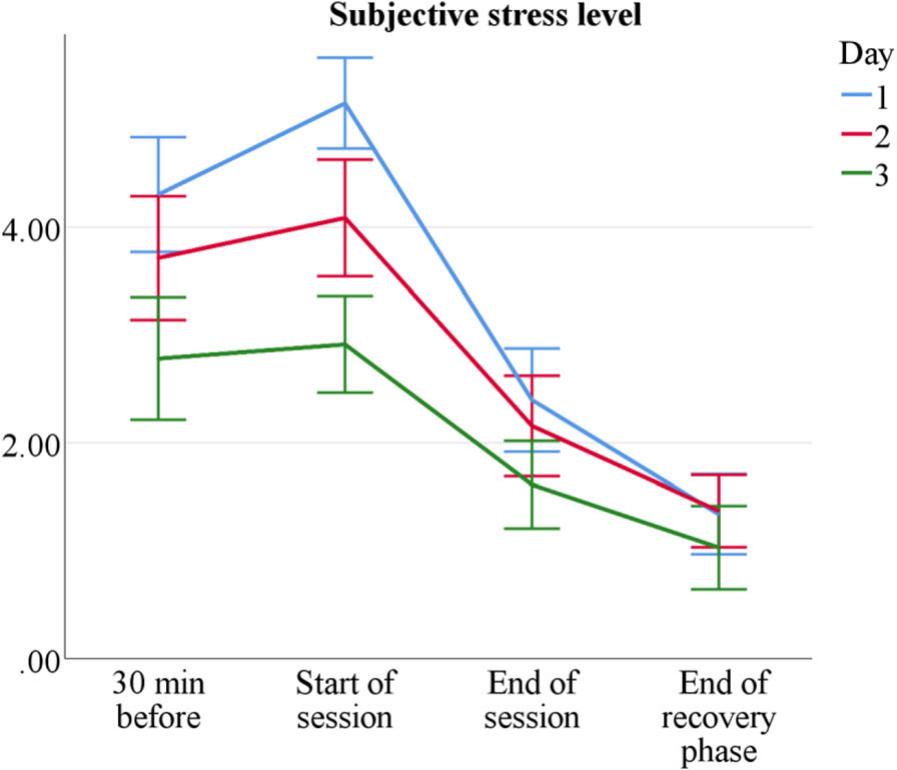
Mean subjective stress level by measurement point and session. Scale range 0–10. *n* = 60. Error bars show ±1.96 *SE*

Table 1 shows the RM-ANOVA results for all psychological stress measures. Tutors report medium subjective stress levels of *M* = 3.48 (*SD* = 2.36) 30 min and *M* = 3.80 (*SD* = 2.42) immediately before session start. After session completion, they report lower values, *M* = 1.97 (*SD* = 1.84), and *M* = 1.22 (*SD* = 1.41) after recovery. Stress levels progressively decrease over course days, as shown in Fig. 2. They are higher for sonography tutors than for AaL^*plus*^ tutors, overall *M* = 3.23 (*SD* = 2.33) vs. overall *M* = 2.21 (*SD* = 2.19), and higher for novice than for senior tutors, *M*_Δ_ = 0.70. The general stress pattern is similar but not equal over conditions, as indicated by significant interaction effects: On earlier days, with low experience, and in sonography tutors, stress values start higher but decline more steeply (see Table 2). Taken together, tutors experience moderate stress levels and recover well after sessions.

**Table 1:** Test statistics for RM-ANOVAs (psychological measures, main and first order interaction effects)

Factors	VAS-Stress			Positive Affect			Negative Affect		
	*F*	*df*	*p*	*F*	*df*	*p*	*F*	*df*	*p*
Time	98.00	2.44, 136.62	<.001	137.67	2, 112	<.001	75.44	1.66, 92.97	<.001
Time*Type	8.04	2.44, 136.62	<.001	4.10	2, 112	.02	2.12	1.66, 92.97	.13
Time*Exp	8.00	2.44, 136.62	<.001	1.52	2, 112	.22	5.18	1.66, 92.97	.01
Day	26.24	2, 112	<.001	11.07	2, 112	<.001	5.29	1.56, 87.26	.01
Day*Type	1.83	2, 112	.17	1.00	2, 112	.37	0.11	1.56, 87.26	.85
Day*Exp	0.68	2, 112	.50	1.18	2, 112	.33	0.33	1.56, 87.26	.67
Time*Day	7.68	4.87, 272.79	<.001	1.30	4, 224	.27	4.68	3.18, 178.26	.003
Type	0.92	1, 56	.002	0.39	1, 56	.54	0.66	1, 56	.42
Exp	4.04	1, 56	<.05	2.18	1, 56	.15	1.24	1, 56	.27
Type*Exp	3.42	1, 56	.07	0.07	1, 56	.79	0.97	1, 56	.33

*Note*. *F*-test statistics of main and first order interaction effects with Visual Analogue Scale (VAS-Stress) scores, Positive Affect, and Negative Affect as respective dependent variables. Independent variables: *Time* Point of measurement, *Day* Day of measurement, *Type* tutor type (AaL^*plus*^, *n* = 36, vs. sonography, *n* = 24), *Exp* tutor experience (senior, *n* = 32 vs. novice tutors, *n* = 28). Mauchly tests for sphericity: VAS: *W*_Time_ = .62, *p* < .001, *W*_Day_ = 92, *p* = .09, *W*_Time*Day_ = .31, *p* < .001; Positive Affect: All *W*’s > .81, *p*’s > .27; Negative Affect: All *W*’s < .63, *p*’s < .001. Within-subject factor statistics corrected for nonsphericity (where appropriate) using the Huynh-Feldt procedure, *F F*-statistic, *df* (corrected) numerator and denominator degrees of freedom, *p p*-value

**Table 2:** VAS-Stress means over conditions

	Day 1	Day 2	Day 3
AaL^*plus*^			
30 min before	3.76 ± 2.53	3.06 ± 2.55	2.49 ± 2.38
Start of session	3.96 ± 2.27	3.12 ± 2.50	1.93 ± 1.75
End of session	1.77 ± 1.89	1.66 ± 1.46	1.29 ± 1.44
End of recovery phase	1.00 ± 1.35	1.20 ± 1.22	1.28 ± 1.72
Sonography			
30 min before	4.88 ± 1.77	4.29 ± 1.75	3.01 ± 1.96
Start of session	6.25 ± 1.50	4.97 ± 1.79	3.80 ± 2.04
End of session	3.08 ± 2.05	2.66 ± 2.18	1.95 ± 1.69
End of recovery phase	1.54 ± 1.45	1.51 ± 1.40	0.75 ± 1.00

*Note. n*_AaL_ = 36, *n*_Sono_ = 24. Stress level means and standard deviations by tutor type, session, and measurement point

**Fig. 3:**
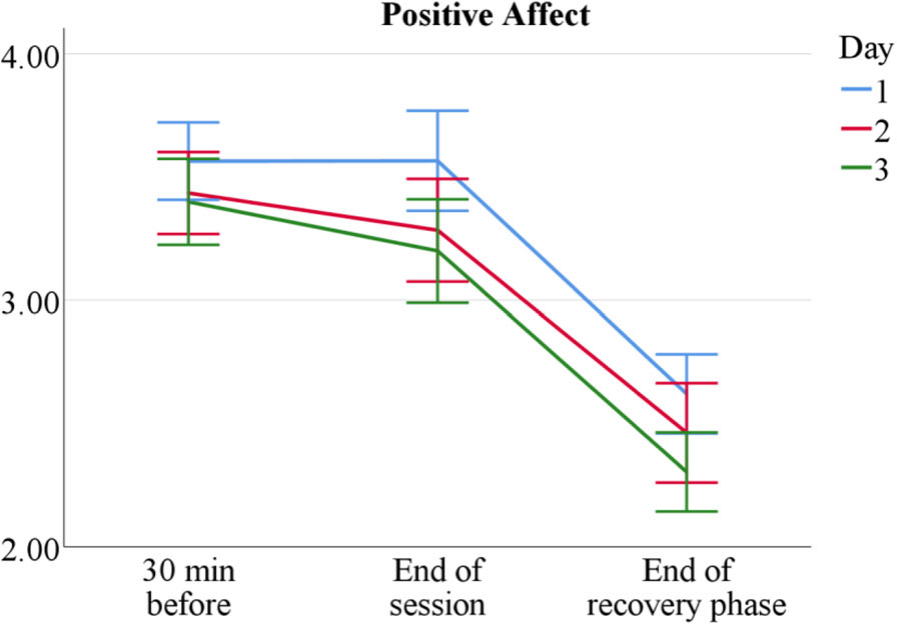
Mean positive affect by measurement point and session. *n* = 60. Scale range 1–5. Error bars show ±1.96 *SE*

Tutors’ positive affect is generally high, both 30 min before the start of the sessions, *M* = 3.45 (*SD* = 0.65), and after the end of the sessions, *M* = 3.36 (*SD* = 0.80). However, positive affect drops to *M* = 2.48 (*SD* = 0.71) after the recovery phase, presumably due to lower activation and wakefulness. It also decreases slightly, but significantly over measurement days, as shown in Fig. 3 and Table 3. Nevertheless, these decreases are moderate and senior tutors report the same amount of positive affect as novice tutors. Sonography tutors do not differ systematically in positive affect from AaL^*plus*^ tutors, except for a slightly more pronounced decrease after recovery.

**Table 3:** PA means over conditions

	Day 1	Day 2	Day 3
AaL^*plus*^			
30 min before	3.44 ± 0.59	3.43 ± 0.65	3.35 ± 0.73
End of session	3.61 ± 0.78	3.31 ± 0.83	3.25 ± 0.85
End of recovery phase	2.66 ± 0.60	2.58 ± 0.81	2.53 ± 0.62
Sonography			
30 min before	3.68 ± 0.68	3.43 ± 0.65	3.45 ± 0.58
End of session	3.51 ± 0.79	3.25 ± 0.74	3.15 ± 0.71
End of recovery phase	2.54 ± 0.74	2.32 ± 0.72	2.05 ± 0.63

*Note. n*_AaL_ = 36, *n*_Sono_ = 24. Positive affect (PA) means and standard deviations by tutor type, day, and measurement point

**Fig. 4:**
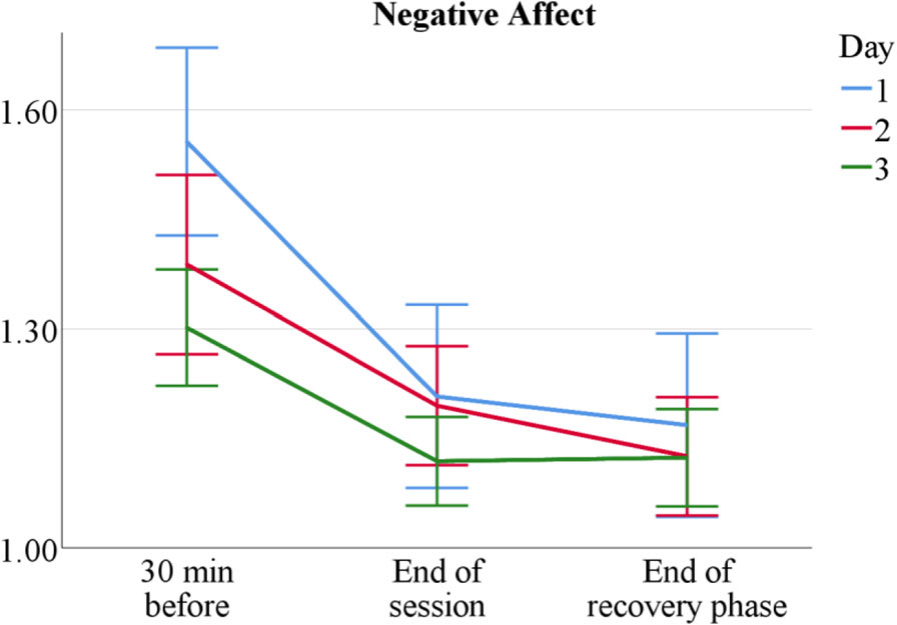
Mean negative affect by measurement point and session. *n* = 60. Scale range 1–5. Error bars show ±1.96 *SE*

Tutors report low negative affect that decreases over a course’s measurement period: *M* = 1.41 (*SD* = 0.45) before the start of sessions, *M* = 1.18 (*SD* = 0.35) after sessions, and *M* = 1.15 (*SD* = 0.36) after recovery phases. Furthermore, it declines over measurement days, as shown in Fig. 4 and Table 4. Tutor experience does not generally influence negative affect levels, with the exception of the first measurement 30 min before session start, where novice tutors show higher levels than senior tutors; *t*(178) = 2.89, *p* = .006, see Table 1: no *Exp* main effect but significant interaction effect Time*Exp. Taken together, even though tutors demonstrate moderate levels of psychological (VAS-Stress) and physiological arousal, they experience only very limited negative affect.

**Table 4:** NA means over conditions

	Day 1	Day 2	Day 3
AaL^*plus*^			
30 min before	1.56 ± 0.62	1.42 ± 0.59	1.30 ± 0.36
End of session	1.21 ± 0.56	1.19 ± 0.35	1.16 ± 0.27
End of recovery phase	1.22 ± 0.59	1.17 ± 0.38	1.21 ± 0.32
Sonography			
30 min before	1.54 ± 0.29	1.34 ± 0.22	1.30 ± 0.24
End of session	1.21 ± 0.34	1.20 ± 0.24	1.08 ± 0.15
End of recovery phase	1.17 ± 0.48	1.08 ± 0.14	1.04 ± 0.10

*Note. n*_AaL_ = 36, *n*_Sono_ = 24. Negative affect (NA) means and standard deviations by tutor type, day, and measurement point

### Physiological stress

Tutors’ absolute salivary cortisol levels were moderate, ranging from 0.15 to 12.86 ng/ml, *M* = 2.13 (*SD* = 1.72). Careful data screening showed no signs of systematic hypo- or hypercortisolism that would impair interpretability; for instance, only 1.8% of cortisol data were above 8.0 ng/ml. Intra-assay variance was 4.04% on average, inter-assay variance was below 15%. As described above, we applied a HLM to determine individual time-dependent low-activity cortisol reference values and identified a random coefficients model with time as random and fixed factor as the best fitting model, AIC = 727.3, log-likelihood = − 357.7, *df* = 198; predictor statistics *t*_time.fixed_(198) = − 5.79, *p* < .001; *Var*_time.random_ = 0.0038: This model was clearly superior to a random intercept null model, AIC = 753.4, log-likelihood = − 373.7, *df* = 201, ICC = .65; χ^2^(3) = 32.05, *p* < .001. Tutor type was tentatively included as predictor into the analysis, but again removed as it proved nonsignificant, *t*(197) = 1.17, *p* = .24.

Table 5 shows the RM-ANOVA results for all physiological stress measures. Tutors’ overall HPA axis activation (in comparison to individual rest day value) by teaching was low and differed significantly between AaL^*plus*^ tutors, *M* = −.43 (*SD* = 1.82), and sonography tutors, *M* = 0.73 (*SD* = 1.68). Negative net cortisol values do not necessarily imply total relaxation, but lower physiological arousal in comparison to a chosen Sunday, which may be relatively active for some tutors.

**Table 5:** Test statistics for RM-ANOVAs (physiological measures, main and first order interaction effects)

Factors	Net Cortisol			RMSSD		
	*F*	*df*	*p*	*F*	*df*	*p*
Time	14.43	1.79, 99.95	<.001	119.45	2.52, 140.97	< .001
Time*Type	1.84	1.79, 99.95	.17	3.66	2.52, 140.97	.02
Time*Exp	0.98	1.79, 99.95	.37	0.84	2.52, 140.97	.46
Day	5.08	1.58, 88.47	.01	7.78	1.62, 90.91	.002
Day*Type	1.40	1.58, 88.47	.25	2.89	1.62, 90.91	.07
Day*Exp	0.95	1.58, 88.47	.37	0.10	1.62, 90.91	.86
Time*Day	2.41	3.33, 186.41	.06	0.79	2.39, 134.00	.48
Type	11.06	1, 56	.002	0.00	1, 56	.95
Exp	0.79	1, 56	.38	0.81	1, 56	.37
Type*Exp	1.03	1, 56	.32	0.96	1, 56	.33

*Note*. *F*-test statistics of main and first order interaction effects with net cortisol and root mean square of successive differences (RMSSD) in interbeat intervals as respective dependent variables. Independent variables: *Time* Point of measurement, *Day* Day of measurement, *Type* tutor type (AaL^*plus*^, *n* = 36, vs. sonography, *n* = 24), *Exp* tutor experience (senior, *n* = 32 vs. novice tutors, *n* = 28). Mauchly tests for sphericity: Net cortisol: All *W*’s < .79, *p*’s ≤ .001; RMSSD: All *W*’s < .68, *p*’s < .001. Within-subject factor statistics corrected for nonsphericity (where appropriate) using the Huynh-Feldt procedure, *F F*-statistic, *df* (corrected) numerator and denominator degrees of freedom, *p p*-value

**Fig. 5:**
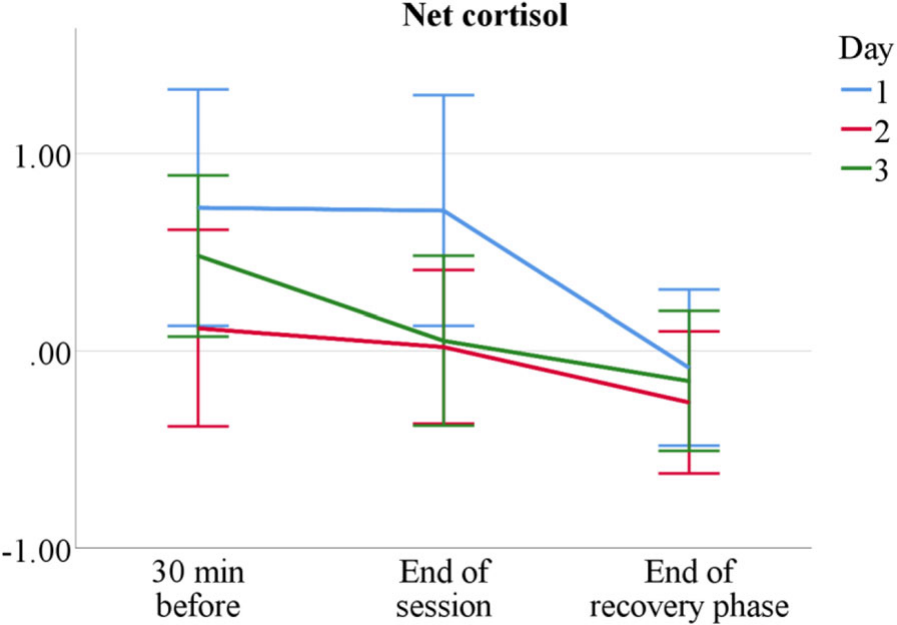
Mean net salivary cortisol by measurement point and session in Δng/ml. *n* = 60. Error bars show ±1.96 SE

Similar to the psychological indices, stress measured by salivary cortisol generally declines over the measurement period. It is higher before the start of the session and slowly drops until its end, except for the first day, on which cortisol levels only fall after the recovery phase. Figure 5 visualises this pattern; Table 6 reveals more details about the differences between tutor types. On day 3, sonography tutors show relatively high cortisol values, presumably due to an additional free practice session offered to course participants on that day, which requires tutors to be present at least one hour earlier for support and assistance. Sonography tutors’ raised cortisol levels after the first course day can be explained by taking the HPA axis’s delayed reactivity into account: They match tutors’ high subjective stress directly before session start on that day (Table 2). Taken together, sympathetic stress levels are altogether moderate; they are mostly lower than on a typical rest day for AaL^*plus*^ tutors and slightly higher for sonography tutors.

**Table 6:** Net cortisol means over conditions

	Day 1	Day 2	Day 3
AaL^*plus*^			
30 min before	0.34 ± 2.10	−0.38 ± 2.00	−0.42 ± 1.57
End of session	−0.04 ± 2.50	−0.63 ± 1.51	− 0.70 ± 1.59
End of recovery phase	−0.49 ± 1.78	− 0.73 ± 1.51	−0.78 ± 1.49
Sonography			
30 min before	1.14 ± 2.54	0.64 ± 1.80	1.40 ± 1.54
End of session	1.53 ± 1.92	0.68 ± 1.52	0.80 ± 1.76
End of recovery phase	0.32 ± 1.13	0.19 ± 1.30	0.49 ± 1.18

*Note. n*_AaL_ = 36, *n*_Sono_ = 24. Net cortisol means and standard deviations by tutor type, day, and measurement point

**Fig. 6:**
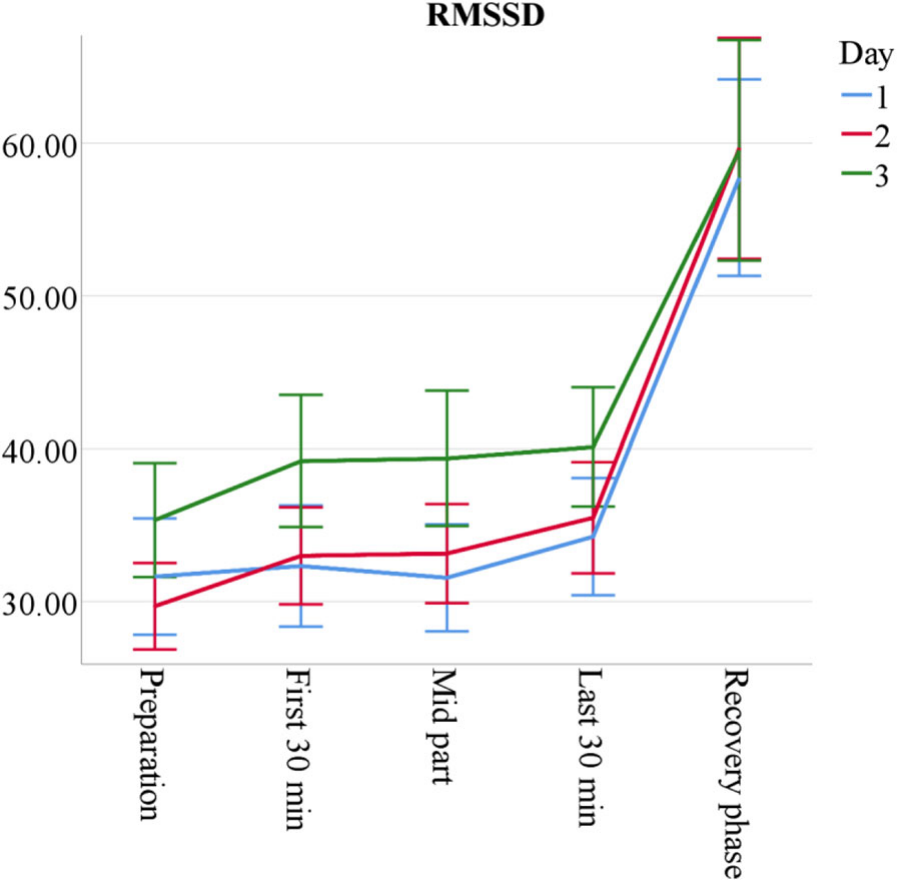
Mean root mean square of successive differences in interbeat intervals (RMSSD) by measurement point and session in ms. *n* = 60. Error bars show ±1.96 SE

The RMSSD, as main parameter for HRV and thus parasympathetic activity, revealed moderate overall physiological stress that decreases over the measurement period. The RMSSD’s total mean (recovery phase excluded) was *M* = 34.82 (*SD* ± 14.68); only two participants showed an individual mean lower than 20. For comparison, a typical RMSSD rest value in healthy populations is 42 (*SD* = 15) [109]. In a sample of first semester medical students, *M*_age_ = 19.80 (*SD*_age_ = 2.12), Huhn et al. [110] found an RMSSD of *M =* 59.45 (*SD* = 35.31) in a rest condition, *M* = 47.21 (*SD* = 29.68) during a seminar, and *M* = 15.47 (*SD* = 9.25) during an oral examination. Therefore, tutors’ physiological stress in courses was slightly higher than in typical attentive states, but clearly lower than in specific high demand situations. Figure 6 visualises the RMSSD profiles found in this study; Table 7 reveals detailed data for AaL^*plus*^ and sonography tutors. On average, the two tutors types do not differ, but a significant interaction effect (Time*Type) indicates a stronger recovery of sonography tutors after sessions. Day 3, however, is an exception from this trend, due to course structure variations that reflect in the data: On this day, AaL^*plus*^ tutors hosted a problem-based learning session, which requires a more passive and laid-back facilitator role except for the session closing; conversely, sonography tutors had less time pressure but extended session duration because of the additional free practice hour taking place on that day. Furthermore, sonography tutors showed higher baseline RMSSD values at the initial measurement point, *M*_Sono_ = 65.63 (*SD*_Sono_ = 32.67), *M*_AaL_ = 50.66, (*SD*_AaL_ = 26.01); *F*(1, 56) = 4.32, *p* = .04. The disappearance of this difference implies a larger decrease of parasympathetic arousal during teaching. Taken together, these results indicate higher stress in sonography tutors when compared to AaL^*plus*^ tutors.

**Table 7:** RMSSD means over conditions

	Day 1	Day 2	Day 3
AaL^*plus*^			
30 min before	30.13 ± 14.72	33.46 ± 11.85	37.27 ± 15.43
First 30 min of session	31.49 ± 13.80	30.30 ± 10.38	41.68 ± 18.28
Mid part of session	34.55 ± 15.15	32.43 ± 10.55	43.87 ± 19.89
Last 30 min of session	37.62 ± 16.32	36.10 ± 13.20	37.64 ± 15.42
Recovery phase	53.79 ± 21.84	52.85 ± 22.43	63.52 ± 32.95
Sonography			
30 min before	32.98 ± 14.57	26.29 ± 10.01	33.60 ± 12.25
First 30 min of session	32.96 ± 17.28	35.56 ± 14.52	36.96 ± 13.35
Mid part of session	28.79 ± 10.24	33.95 ± 14.83	35.12 ± 10.92
Last 30 min of session	31.18 ± 12.08	35.04 ± 15.56	42.28 ± 14.52
Recovery phase	61.96 ± 28.34	67.90 ± 34.00	55.75 ± 18.83

*Note. n*_AaL_ = 36, *n*_Sono_ = 24. Root mean square of successive differences (RMSSD) means and standard deviations by tutor type, day, and measurement point

All other HRV indices display similar profiles and closely mirror the results obtained for the RMSSD: parasympathetic activity slowly increases both over single course sessions, with a strong rise during the recovery phase, as well as over measurement days, especially on day 3. A comprehensive overview on all HRV data can be found in Additional file 2: Table S2 within the online supplementary material.

### Correlations between personality and stress measures

To determine the influence of personality on individuals’ stress, we sequentially included the assessed personality variables as covariates into the RM-ANOVAs reported above. The correlations with the different stress measures thus obtained are adjusted for measurement point, measurement day, tutor type, and tutor experience, and shown in Table 8.

**Table 8:** Correlations of personality and adjusted stress measures

	VAS-Stress	Positive Affect	Negative Affect	Cortisol	RMSSD
Personality: BFI-K					
Extraversion	−.16	.31*†	−.44***†	−.11	.32*†
Neuroticism	.26*	−.06	.28*	−.11	.00
Conscientiousness	−.18	.22	−.29*	−.14	.14
Agreeableness	.03	.36**†	−.06	.07	−.13
Openness	−.04	−.01	−.09	−.08	.07
Attachment style: AAS					
Depend	−.22	.14	−.24	−.05	−.02
Anxiety	.27*	−.11	.19	−.10	.00
Close	−.30*	.13	−.28*	.05	−.02
Resilience: RS 13					
Competence	−.31*	.30*	−.48***†	.04	.06
Acceptance	−.35**†	.11	−.37**†	.10	−.13
Perfectionism: SAPS-R					
Standards	−.08	.03	−.24	.04	.00
Discrepancy	.25	−.18	.18	−.01	−.10

*Note*. Pearson-correlations of personality and stress measures, adjusted for measurement times, tutor type and experience. *VAS* Visual Analogue Scale, *RMSSD* root mean square of successive differences in interbeat intervals

*n* = 60. * *p* < .05. ** *p* < .01. *** *p* < .001. † significant after Bonferroni-Holm-corrections for multiple comparisons [114]

Extraversion was correlated with high positive and low negative affect, which is not surprising, given the interactive and interpersonal nature of teaching demands. Furthermore, we found extraversion to be the only personality trait correlated with a physiological stress index, the RMSSD. Agreeableness was positively correlated with reported positive affect. Resilience was a protective factor against subjective stress and negative affect. Other personality traits, namely conscientiousness, openness, attachment style, and perfectionism, did not or only in tendency show correlations with stress measures.

The tutors evaluated their courses’ quality generally as high, *M* = 7.76 (*SD* = 1.36). Overall course quality ratings did not differ between senior and novice tutors, *F*(1, 56) = 1.42, *p* = .24; however, they were significantly correlated with subjective stress measures, *r*_VAS_ = −.20, *r*_PA_ = .34, *r*_NA_ = −.29, but not with physiological stress measures, *r*_Cortisol_ = −.09, *r*_RMSSD_ = .02*.*

### Stressors and life events

Three category systems resulted from inductive content analyses [108] of the questionnaires’ free entry fields: Stressors during AaL^*plus*^ (3 × 36 entries) and sonography courses (3 × 24 entries) as well as tutors’ general everyday life stressors (60 entries). Multiple mentions per entry field were possible.

Table 9 lists the resulting categories of stressors during sessions along with category descriptions, mentions counts and exemplary mentions. Stressors found in both courses were time pressure, leadership role demands, uncertainties in medical knowledge, as well as single participants’ characteristics; however, time pressure was more prevalent in sonography courses and leadership role demands in AaL^*plus*^ courses. Furthermore, organisational difficulties (like late arrivals or technical problems) and coordination within the teaching team posed additional challenges for AaL^*plus*^ tutors, whereas sonography tutors had to handle bad sonographic conditions in some ultrasound models. After 41 (38%) AaL^*plus*^ sessions and 23 (32%) sonography sessions, tutors reported no stressors.

**Table 9:** Category system of stressors during sessions

AaL^*plus*^	
Group characteristics (13)	Passive or sluggish groups, loud and inattentive groups, or strong differences in prior knowledge. *“Very silent participants, hardly any volunteers for history taking exercises.”*
Team teaching coordination (13)	Teaching partner shows low involvement, is too dominant, or omits preparatory meetings. *“I took on the largest part of teaching because I felt my partner is not prepared.”*
Organisational conditions (13)	Last-minute preparations, especially when devices do not function properly, or late arrivals of standardised patient actors or students. *“The projector’s sound did not work; we then showed the video on a laptop computer instead.”*
Leadership role demands (10)	Group instruction (6 mentions), speaking in front of participants, or giving feedback. *“Keeping an eye on participants: who needs further assistance and is too shy to ask.”*
Time pressure(9)	Lack of time both during a session and due to the additional workload for preparation and teaching. *“Too little time at the end of the session.” “Time lost due to teaching: I have an exam on Friday.”*
Uncertainties in medical knowledge (9)	Limits of own medical knowledge, mostly encountered when preparing and giving presentations. *“A student’s question about thrombosis prophylaxis which I could not answer.”*
Supervision (5)	Routine supervisions by faculty staff member. *“Supervision was stressful.” “The supervision made me nervous, though mostly before the start of the session.”*
Participant characteristics (3)	Single participants’ character traits or behaviour. *“One student was very dominant, wants to convey extra knowledge but goes beyond the scope of the course; the group was partly irritated.”*
Personal discomfort (2)	Hunger or minor illness. *“Dry cough.”*
Total mentions: 77; “None” or no mention: 41	
Sonography	
Time pressure (16)	Tight schedule and extensive subject matter, little buffer for delays or participants’ knowledge gaps. *“Much content, little time. I got delayed at the gall bladder.” “Time management and order.”*
Ultrasound difficulties (9)	Bad sonographic conditions that complicate detecting structures and locating standard scan planes. *“Both ultrasound demonstrations were poor (overlaying intestinal gases).”*
Poor participant performance (9)	Unprepared and unskilled participants. *“Participants need a lot of assistance”* *“Participants were not properly prepared and had forgotten the last days’ contents.”*
Leadership role demands (7)	Difficulties in motivating participants and directing group processes. *“Keeping participants attentive so that nobody gets bored.” “When students could not answer theoretical questions.”*
Uncertainties in medical knowledge (5)	Limits of own medical knowledge, i.e. when identifying structures or assisting participants. *“Needed help to adjust the device.” “Twice, I did not know how to concretely assist participants.”*
Participant characteristics (4)	Single participants’ character traits or behaviour. *“Problems of understanding with a foreign student.” “A student’s constant private conversations”.*
Group characteristics (3)	Noisy, inattentive, or heterogeneous groups. *“Participants vary a lot in terms of prior knowledge.”*
Personal discomfort (3)	Minor illness. *“I had a cold and was hoarse.”*
Total mentions: 56; “None” or no mention: 23	

*Note*. Resulting categories of stressors during sessions, based on 108 free entry fields for AaL^*plus*^ tutors and 72 for sonography tutors. Respective numbers of mentions within each category are shown in parentheses, exemplary mentions in italics

Table 10 presents content analysis results for general stressors. On average, tutors report about two relevant general stressors, mostly related to their academic life: Studying for exams, teaching and preparing peer teaching, and working for their doctorate are the most prevalent stressors. Beyond that, the other categories provide insight into further daily challenges encountered by tutors.

**Table 10:** Category system of general stressors

Studies and exams (29)	Study workload, clerkships, compulsory courses, and upcoming examinations. *“Lots of compulsory courses.” “Exam week in both natural medicine and emergency care.”*
Teaching and preparation (18)	Preparing and holding courses, as well as the necessary time for preparation. *“AaL* ^plus^ *sessions 3x/week.” “The preparation for the tutorial courses was very demanding.”*
Doctorate (13)	Applying for and preparing a doctorate, research and laboratory work, and writing a thesis. *“Much work in the lab, especially a presentation of the last months’ results.”*
Part-time jobs (12)	Mostly as undergraduate assistants or shifts at hospital wards. *“Additional night shifts, altogether about 4x last month; at times only two hours of sleep.”*
Travel, family visits and Erasmus stays (8)	Larger travels, Erasmus study stays abroad, family visits. *“Return from my Erasmus stay.” “First time at home for Christmas for three years.”*
Housing search and moving (8)	Flat viewing and moves. *“I had to move houses, rent a transporter, carry furniture, arrange the rental contract, and register at the residents’ office – all parallel to the sonography course.”*
Illness (own and family members’) (6)	Health restrictions and worries. *“I had a serious flu.” “My mother’s illness. Due to her language barriers, I had to help her with her rehab application.”*
Engagements (5)	Engagement in social projects or the faculty’s student body. *“Tasks in the student council.”*
Other (14)	Some mentions could not be summarised in any category, such as selection interviews for scholarships, personal conflicts, break-ups, imminent deadlines, or sports competitions.
Unspecific (10)	General stress without a specified stressor. *“Psychological stress due to life circumstances.”*
Total mentions: 123; “None” or no mention: 13	

*Note*. Resulting categories of general everyday life stressors, based on 60 free entry fields. Respective numbers of mentions within each category are shown in parentheses, exemplary mentions in italics

Taken together, tutors experience a wide array of stressors. However, specific numbers differ widely: Most tutors report multiple stressors, yet a noticeable proportion mentions no or only limited stressors.

## Discussion

### Principal findings

The tutors examined in this study are mostly outgoing, secure, and resilient young adults (see Table 1). Except for slightly higher pressure to perform and lower social overload, their self-reports on chronic stress resemble general population values; hence, the participating tutors appear to dispose of sufficient resources, including leadership experience, to meet the demands and challenges of teaching fellow students. We further showed that, on a correlational level, the tutors’ above-average extraversion and resilience was a protective factor. Even though this study did not focus on mental health or group comparisons, the results do not indicate the heightened psychopathological burden commonly found in medical student populations [25–27]. This is not altogether surprising, given that Heidelberg and numerous other medical schools stringently select students with outstanding academic and social abilities for their tutor programmes and extensively prepare them for their tutoring activities in special training [17].

The comparison of two tutorial course types shows both similarities and differences in tutors’ stress experience. Sonography tutors are more stressed than AaL^*plus*^ tutors (see Tables 2 and 6) and, as expected, novice tutors are more than senior tutors (see Tables 1 and 5); however, despite occasional stress peaks, tutors generally experience teaching as stimulating and only moderately stressful (see Figs. 2, 3, 4, 5 and 6).

All employed stress measures revealed similar profiles: Psychological and physiological stress are moderately high before the start of the session, decline to its end and subside after the recovery phase; moreover, stress decreases over course days. This corresponds to a normal and healthy stress response cycle; especially the cessation of remaining arousal during recovery indicates sufficient and successful coping [28, 80]. These findings are independent from course type or tutor experience, with minor exceptions on the third measurement day: Sonography tutors’ cortisol levels slightly increase, probably due to additional teaching demands, whereas AaL^*plus*^ tutors show noticeably high RMSSD values on that day, which is likely to reflect their more passive, moderating role during problem-based learning sessions.

Differences in demands between course types show not only in quantitative data but also in tutors’ qualitative statements. AaL^*plus*^-specific stressors encompass difficult group dynamics or team-teaching coordination problems; within sonography courses, tight schedules and extensive teaching contents pose the largest challenges, especially when course participants perform poorly or sonographic conditions are unfavourable. Teaching-related stressors found in both course types are time pressure, leadership position demands, and participant as well as group characteristics.

However, even though tutors name a multitude of course-related stressors, all participating tutors, independent of course type or tutor experience, report high positive affect that only declines very slightly over the measurement period. This indicates that positive emotions during teaching do not decline through routine, waning interest, or other habituation effects. Furthermore, the tutors’ self-rated course quality is generally high. This indicates a constantly high personal involvement which does not diminish over time, for instance due to habituation, routine, or insufficient stimulation.

Taken together, even though peer teaching poses noticeable demands on tutors, it does not seem to raise stress to levels that endanger students’ mental health [26, 35, 38, 111]. To the contrary, the results suggest that tutors experience a well-matched balance of environmental challenges and individual capacities, which is known to have potentially stimulating, engaging, and generally beneficial effects on learning and performance [32–34]. This demonstrates the possibility of creating peer teaching programmes that are viable, beneficial, and healthy not only for participating students, but also for tutors themselves, at least given an adequate selection and training process.

### Strengths and limitations

This study’s multidimensional assessment, utilising a variety of psychophysiological stress indices at multiple measurement points and days in a naturalistic setting, provides a comprehensive and valid view on tutors’ stress. All stress markers possess good to excellent quality criteria; moreover, known variations in course agendas, for instance at the third day, are closely reflected in stress measures, which further corroborates measurement validity. Cortisol measures, however, show relatively large standard errors, potentially due to the moderate stress levels that may not have sufficed to evoke a stronger HPA axis response. Given the large number of repeated measures, the study’s sample size (*n* = 60) was sufficient for all analyses. The pseudonymisation of participant data protected from social desirability effects.

The online measurement of HRV data allowed close tracking of individual stress profiles. Unfortunately, the study design did not permit assignment of single events or specific situations to variations in these profiles; therefore, we could only determine the average individual parasympathetic activity during teaching. However, tutors’ qualitative statements close this gap by providing detailed insight into the nature and frequency of stressors they experience in teaching and their everyday life.

Qualitative analyses are interpretative by nature: Although data categorisation procedures are rule- and theory-guided, it is possible that different analysis considerations yield different results. Moreover, only one author performed the analyses in this study. Nonetheless, due to the narrow and clearly circumscribed research questions and the unambiguous participant answers, this interpretative element was minimised.

As the study participation rates of AaL^*plus*^ (68%) and sonography (57%) tutors are very high (63% overall), this study allows valid assertions about stress and stressors in these two structured tutorial programmes. However, medical faculties’ organisational structures and fields of application for peer teaching are diverse, which is why other tutor programmes come with potentially different requirements and demands on tutors that may result in different stressors and stress profiles [17].

### Directions for future research and application

Past research on peer assisted learning has focussed on exploring and establishing diverse areas of application, as well as determining their feasibility and efficacy. As a next step, researchers should additionally take health and stress in different tutor samples into account. Chronic stress and its associated health problems are highly prevalent among physicians and often start to manifest in study years [40–42]; moreover, especially the combined burdens of frequent, multiple stressor exposure seem to promote mental illness in medical students [112, 113]. The results presented in this study may serve as a benchmark against which quantitative and qualitative stress measures from future studies can be compared.

Although the tutors examined in this study cope successfully, differences in stress levels exist, depending on course type and tutor experience. Experience and habituation influence stress and negative affect, but not positive affect. We further showed that even well-structured peer-teaching courses induce numerous additional stressors, that in other contexts or for some individuals may exceed a healthy coping capacity [28, 44]. Moreover, we found that low stress was significantly correlated with higher course quality, at least on a subjective level. For these reasons, curriculum designers and staff responsible for peer teaching programmes should mind the potential burdens of tutoring and, if necessary, make efforts to mitigate stress and initiate adequate prevention strategies. A more precise knowledge of stressors frequently encountered by peer teachers, as presented in this study, can support staff in achieving this objective. For instance, in our tutor training, we prepare tutors both in terms of medical-technical as well as leadership skills, such as handling time pressure, group dynamics, difficult participant characteristics, and other tutor role demands. In addition to tailored training, tutor supervision may specifically address these frequent stressors. Moreover, health and coping can already be taken into account when recruiting new tutors: Our results suggest that extraversion and general resilience are two suitable selection criteria.

## Conclusion

Structured peer-led tutorial courses, an effective, feasible, and cost-efficient peer teaching method, put various demands on student tutors, such as time pressure, leadership, and course preparation. Nonetheless, tutors display various indicators of successful coping: moderate psychological and physiological stress, consistently high positive affect without indicators for loss of interest, and good recovery after sessions. Tutors’ actual stress levels partly depend on experience and course characteristics. They self-evaluate their courses positively. These results corroborate the viability and success of current peer-teaching programmes from the tutors’ perspective.

This study presents a comprehensive overview on peer tutors’ stress and stressors: an extensive quantification of psychophysiological stress levels provides a benchmark for future research, a detailed account of frequent stressors contributes to the development of better peer teaching programmes, tutor qualification training, or stress prevention strategies.

## Additional files

Additional file 1: **Table S1.** Sample characteristics and comparisons to norm samples. Table S1 presents descriptive data about personality (BFI-K), attachment style (AAS), resilience (RS 13), perfectionism (SAPS-R) and chronic stress (TICS) in the tutor sample and norm samples. It further shows sample comparison test statistics and descriptive data on tutors’ lifestyle (physical activity, relationship status, prior teaching and leadership experience, sleep per night, substance use, and weekly time for studies, hobbies, and other activities). (DOCX 25 kb)

Additional file 2: **Table S2**. Heart rate measures in the different course sections on all three measurement days. Table S2 gives a comprehensive overview on all heart rates measures and derived indices on all measurement occasions. The reported data comprise time domain measures (mRR, RMSSD, heart rate, SDNN, and pNN50) as well as frequency domain measures (VLF, LF, and HF component, LF/HF ratio) obtained with autoregressive spectral modelling. (DOCX 20 kb)
