# Supplementary material for: Stress and stressors of medical student near-peer tutors during courses: a psychophysiological mixed methods study
Source: BMC Med Educ. 2019 Apr 2;19:95. doi: 10.1186/s12909-019-1521-2 (PMC6444608; doi:10.1186/s12909-019-1521-2)
Supplement: Supplementary file 1 — Table S1. Sample characteristics and comparisons to norm samples. Table S1 presents descriptive data about personality (BFI-K), attachment style (AAS), resilience (RS 13), perfectionism (SAPS-R) and chronic stress (TICS) in the tutor sample and norm samples. It further shows sample comparison test statistics and descriptive data on tutors’ lifestyle (physical activity, relationship status, prior teaching and leadership experience, sleep per night, substance use, and weekly time for studies, hobbies, and other activities). (DOCX 25 kb) [file 12909_2019_1521_MOESM1_ESM.docx]

| Table S1 | | | | | | | | | | |
| --- | --- | --- | --- | --- | --- | --- | --- | --- | --- | --- |
| *Sample characteristics and comparisons to norm samples* | | | | | | | | | | |
|  |  | Tutor sample | |  | Norm samples^1^ | |  | Samples comparison^2^ | | |
| Variable | | *M* | *SD* |  | *M* | *SD* |  | *t* | *p* | *d* |
| Personality: BFI-K     Extraversion     Neuroticism     Conscientiousness     Agreeableness     Openness | | 3.97  2.79  4.06  3.16  4.04 | 0.75  0.99  0.71  0.79  0.66 |  | 3.48  2.88  3.53  3.02  3.96 | 0.87  0.77  0.69  0.73  0.62 |  | 5.06  0.70  5.78  1.37  0.94 | <.001  .48  <.001  .18  .35 | 0.56  0.77 |
| Attachment Style: AAS     Depend     Anxiety     Close | | 4.10  1.94  4.21 | 0.64  0.76  0.76 |  | 3.72  2.11  3.93 | 0.69  0.74  0.70 |  | 2.85  1.73  4.60 | .006  .09  <.001 | 0.55 |
| Resilience: RS 13     Competence     Acceptance | | 5.81  5.06 | 0.54  0.97 |  | 5.33  5.50 | 1.00  1.00 |  | 6.89  3.51 | <.001  .001 | 0.48  −0.44 |
| Perfectionism: SAPS-R     Standards     Discrepancy | | 5.99  2.91 | 0.81  1.35 |  | 6.03  3.34 | 0.91  1.32 |  | 0.38  2.47 | .70  .02 |  |
| Chronic stress: TICS     Work overload     Social overload     Pressure to perform     Work discontent     Excessive demands at work     Lack of social recognition     Social tensions     Social isolation     Chronic worrying     Chronic stress screening scale | | 1.87  1.34  2.05  1.23  0.89  1.04  0.84  1.03  1.49  1.28 | 0.98  0.68  0.58  0.74  0.71  0.80  0.64  0.81  1.08  0.80 |  | 1.50  1.62  1.73  1.14  0.79  1.12  0.95  1.04  1.46  1.20 | 0.80  0.87  0.86  0.66  0.61  0.80  0.65  0.81  0.93  0.69 |  | 2.92  3.19  4.27  0.94  1.09  0.77  1.33  0.10  0.22  0.77 | .005  .002  <.001  .35  .28  .44  .19  .92  .83  .44 | −0.32  0.37 |
| Sports per week (hours) | | 5.21 | 2.87 |  |  |  |  |  |  |  |
| In intimate relationship | | 61.7% | – |  |  |  |  |  |  |  |
| Prior teaching and leadership expercience^3^ | | 3.48 | 1.02 |  |  |  |  |  |  |  |
| Sleep per night (hours) | | 7.09 | 0.79 |  |  |  |  |  |  |  |
| Substance use     Caffeine (cups or glasses per week)     Alcohol (glasses per month)     Cigarettes (packs per month)     Other substances^3^ | | 6.86  13.35  0.04  8.3% | 6.63  12.91  0.16  – |  |  |  |  |  |  |  |
| Weekly time (hours) spent on…     Studies (altogether)     Hobbies     Side job (without tutorial courses)     Extracurricular activities | | 33.43  10.54  3.22  5.26 | 15.11  7.98  4.57  6.66 |  |  |  |  |  |  |  |
| *Note*. Tutor sample *n* = 60. ^1^ Norm samples: BFI-K, 459 students from the universities of Bielefeld and Göttingen, 57% female, *M*_age_ = 25.8 years (*SD* = 5.7) [96]; AAS, representative sample of 2050 German participants, 55.9% female, *M*_age_ = 49.3 years (*SD* = 17.3) [102]; RS 13, mixed data from 731 patients of Jena University Hospital and 1940 participants in a representative survey, 55.6% female, *M*_age_ = 47.2 years (*SD* = 16.6) [100]; SAPS, 749 students from the US South West, 66.8% female, *M*_age_ = 19.6 years (*SD* = 2.0) [97]; TICS, representative sample of 604 participants, *M*_age_ = 23.8 years, (*SD* = 4.3) [103]. ^2^ *t*-test comparisons of peer tutor and norm sample values; Cohen’s *d* effect sizes are given for significant differences after Bonferroni-Holm corrections [111], α_corr_ = .003. ^3^Likert scale ranging from 1 to 6; 1 = “none“, 2 = “little“, 3 = “medium“, 4 = “much“, 5 = “very much“, 6 = “educational training“; ^3^ proportion of answers “rarely” or “occasionally”; mostly cannabis. | | | | | | | | | | |
