# Supplementary material for: Stress and stressors of medical student near-peer tutors during courses: a psychophysiological mixed methods study
Source: BMC Med Educ. 2019 Apr 2;19:95. doi: 10.1186/s12909-019-1521-2 (PMC6444608; doi:10.1186/s12909-019-1521-2)
Supplement: Supplementary file 2 — Table S2. Heart rate measures in the different course sections on all three measurement days. Table S2 gives a comprehensive overview on all heart rates measures and derived indices on all measurement occasions. The reported data comprise time domain measures (mRR, RMSSD, heart rate, SDNN, and pNN50) as well as frequency domain measures (VLF, LF, and HF component, LF/HF ratio) obtained with autoregressive spectral modelling. (DOCX 20 kb) [file 12909_2019_1521_MOESM2_ESM.docx]

| Table S2 | | | | | | |
| --- | --- | --- | --- | --- | --- | --- |
| *Heart rate measures in the different course sections on all three measurement days* | | | | | | |
| Variable |  | 30 min before | First 30 min | Mid part | Last 30 min | Recovery phase |
| Time domain measures | |  |  |  |  |  |
| mRR | Day 1 Day 2 Day 3 | 645 ±106 654 ±101 702 ±101 | 656 ±111 688 ±96 743 ±112 | 690 ±115 708 ±97 760 ±116 | 713 ±110 719 ±97 760 ±118 | 870 ±139 867 ±126 904 ±144 |
| RMSSD | Day 1 Day 2 Day 3 | 31.3 ±14.6 30.6 ±11.6 35.8 ±14.3 | 32.1 ±15.2 32.4 ±12.4 39.8 ±16.5 | 32.3 ±13.6 33.0 ±12.3 40.4 ±17.3 | 35.0 ±15.0 35.7 ±14.1 39.5 ±15.1 | 57.2 ±24.7 58.3 ±28.5 60.4 ±28.2 |
| Heart rate | Day 1 Day 2 Day 3 | 96.8 ±16.2 95.5 ±14.9 88.8 ±12.4 | 95.7 ±16.0 89.6 ±12.0 83.9 ±12.0 | 90.4 ±14.4 87.3 ±11.0 81.4 ±11.4 | 87.2 ±12.8 86.1 ±11.0 81.9 ±11.6 | 63.2 ±21.4 71.4 ±9.7 68.8 ±11.1 |
| SDNN | Day 1 Day 2 Day 3 | 48.6 ±17.4 45.3 ±13.2 50.3 ±14.1 | 48.9 ±16.5 49.0 ±13.0 52.9 ±15.3 | 50.0 ±15.7 50.3 ±12.5 54.0 ±15.5 | 53.8 ±18.4 53.7 ±13.7 54.1 ±14.7 | 63.2 ±21.4 61.2 ±21.9 62.2 ±18.8 |
| pNN50 | Day 1 Day 2 Day 3 | 10.8 ±8.3 9.3 ±7.0 13.2 ±9.9 | 10.8 ±9.3 11.0 ±8.2 16.5 ±11.8 | 11.7 ±9.7 11.7 ±9.4 17.4 ±13.4 | 13.5 ±11.1 13.5 ±10.7 17.2 ±12.2 | 31.3 ±18.6 31.4 ±20.2 34.3 ±20.2 |
| Frequency domain measures | |  |  |  |  |  |
| VLF-component | Day 1 Day 2 Day 3 | 588 ±1222 320 ±377 318 ±183 | 440 ±817 303 ±246 303 ±152 | 446 ±935 294 ±148 317 ±172 | 649 ±1351 352 ±183 335 ±163 | 554 ±996 299 ±228 303 ±173 |
| in % of total power | Day 1 Day 2 Day 3 | 16.1 ±13.5 13.5 ±6.2 11.8 ±2.7 | 14.0 ±12.2 11.7 ±5.1 10.6 ±2.3 | 13.3 ±11.7 11.2 ±2.4 10.6 ±2.6 | 15.7 ±14.3 11.8 ±3.2 11.0 ±2.7 | 11.2 ±10.6 8.3 ±3.3 8.5 ±3.8 |
| LF-component | Day 1 Day 2 Day 3 | 1779 ±997 1493 ±804 1897 ±958 | 1704 ±935 1767 ±794 2005 ±1083 | 1792 ±912 1935 ±900 2114 ±1111 | 1994 ±1131 2173 ±1007 2186 ±1063 | 2248 ±1715 2064 ±1649 1976 ±1172 |
| in % total power | Day 1 Day 2 Day 3 | 67.9 ±13.0 68.9 ±8.2 70.0 ±6.6 | 67.8 ±12.3 70.5 ±7.9 68.4 ±7.9 | 69.6 ±12.3 72.4 ±6.5 68.7 ±9.6 | 67.5 ±14.1 71.9 ±7.2 69.7 ±8.7 | 54.6 ±16.8 54.6 ±15.6 52.8 ±14.7 |
| HF-component | Day 1 Day 2 Day 3 | 457 ±386 399 ±249 530 ±396 | 503 ±462 471 ±415 662 ±549 | 466 ±380 450 ±343 675 ±606 | 544 ±498 518 ±399 631 ±484 | 1479 ±1146 1578 ±1375 1680 ±1544 |
| in % total power | Day 1 Day 2 Day 3 | 16.1 ±7.6 17.7 ±6.5 18.2 ±6.6 | 18.2 ±8.6 17.8 ±6.9 21.1 ±8.7 | 17.1 ±7.8 16.4 ±6.0 20.6 ±10.3 | 16.9 ±7.6 16.4 ±6.4 19.3 ±8.7 | 34.2 ±14.9 37.0 ±16.9 38.7 ±16.1 |
| Total spectral power | Day 1 Day 2 Day 3 | 2824 ±1891 2203 ±1209 2745 ±1442 | 2647 ±1591 2541 ±1240 2970 ±1651 | 2704 ±1597 2680 ±1265 3106 ±1693 | 3187 ±2144 3043 ±1435 3153 ±1553 | 4281 ±2836 3941 ±2745 3958 ±2275 |
| LF/HF-ratio | Day 1 Day 2 Day 3 | 5.11 4.50 4.66 | 4.77 4.73 4.22 | 5.20 5.19 4.59 | 4.97 5.23 4.63 | 2.19 2.41 2.04 |
| *Note*. Complete list of peer tutors’ HRV indices means and standard deviations by measurement day and period. mRR = mean RR-interval (milliseconds), RMSSD = root mean square of successive differences in interbeat intervals (milliseconds), heart rate in beats per minute, SDNN = standard deviation of NN-intervals (milliseconds), pNN50 = percentage of successive differences ≥ 50 ms, VLF, LF, HF: spectral power of very low, low, and high frequency bands, respectively, obtained with autoregressive spectral modelling. *n* = 60. | | | | | | |
